# Delayed and highly specific antibody response to nonstructural protein 1 (NS1) revealed during natural human ZIKV infection by NS1-based capture ELISA

**DOI:** 10.1186/s12879-018-3173-y

**Published:** 2018-06-14

**Authors:** Xiujie Gao, Yingfen Wen, Jian Wang, Wenxin Hong, Chunlin Li, Lingzhai Zhao, Chibiao Yin, Xia Jin, Fuchun Zhang, Lei Yu

**Affiliations:** 1 Guangzhou Eighth People’s Hospital, Guangzhou Medical University, 627 Dongfeng Rd. East, Guangzhou, 510060 China; 2 Viral Disease and Vaccine Translational Research Unit, CAS Key Lab of Molecular Virology and Immunology, Institut Pasteur of Shanghai, Chinese Academy of Sciences (CAS), Shanghai, 200025 China

**Keywords:** Zika virus, Dengue virus, Non-structural protein 1, Antibody response, Cross-reactivity

## Abstract

**Background:**

Zika virus (ZIKV) had spread rapidly in the past few years in southern hemisphere where dengue virus (DENV) had caused epidemic problems for over half a century. The high degree of cross-reactivity of Envelope (E) protein specific antibody responses between ZIKV and DENV made it challenging to perform differential diagnosis between the two infections using standard ELISA method for E protein.

**Methods:**

Using an IgG capture ELISA, we investigated the kinetics of nonstructural protein 1 (NS1) antibody response during natural ZIKV infection and the cross-reactivity to NS1 proteins using convalescent sera obtained from patients infected by either DENV or ZIKV.

**Results:**

The analyses of the sequential serum samples from ZIKV infected individuals showed NS1 specific Abs appeared 2 weeks later than E specific Abs. Notably, human sera from ZIKV infected individuals did not contain cross-reactivity to NS1 proteins of any of the four DENV serotypes. Furthermore, four out of five NS1-specific monoclonal antibodies (mAbs) isolated from ZIKV infected individuals did not bind to DENV NS1 proteins. Only limited amount of cross-reactivity to ZIKV NS1 was displayed in 108 DENV1 immune sera at 1:100 dilution.

**Conclusions:**

The high degree of NS1-specific Abs in both ZIKV and DENV infection revealed here suggest that NS1-based diagnostics would significantly improve the differential diagnosis between DENV and ZIKV infections.

**Electronic supplementary material:**

The online version of this article (10.1186/s12879-018-3173-y) contains supplementary material, which is available to authorized users.

## Background

With the rapid spread of Zika virus (ZIKV) in the Americas in 2015–2016, and its association with fetal skull malformations and neurologic disorders in adults, WHO declared ZIKV a global emergency [1, 2]. A total of 18 imported cases were reported during the year 2016 in China, with 12 cases in Guangdong, south China [3–6]. Historically, Guangdong had sporadic cases of dengue virus (DENV) infection for decades with the serotype 1 of DENV (DENV1) predominant in circulation [7–9]. A major DENV1 outbreak in 2014 in Guangdong had resulted in 13,800 hospitalizations with an estimated 300 cases of severe dengue and five reported deaths [9]. The preexisting DENV immune status in population combined with the risk of endemic spread of ZIKV infection, have raised the question of how to diagnosis each specific infection, and whether disease severity will be altered due to a potential antibody cross-reactivity because of the genetic and structural closeness between these two flaviviruses.

The flavivirus E protein is the main target of human antibody response. It contains three domains, EDI, EDII and EDIII [10]. The high cross-reactivity between ZIKV and DENV E-specific Abs was commonly known because of the similarity of their E proteins in sequence and structure [11–13], especially at an early time point during DENV and ZIKV infections [14, 15]. We have reported that the EDI/EDII binding Abs in sera from ZIKV infected individual peaked and waned earlier than EDIII binding Abs [15], consisting with the knowledge that ZIKV and DENV have a highly conserved fusion loop epitope (FLE) in EDII. The monoclonal antibodies (mAbs) isolated from the plasma cell or memory B cell appearing at early ZIKV infection also showed cross-reactivity with DENV and binding to the EDI/EDII [15].

The non-structural protein 1(NS1) of flavivirus can be released from infected cell to the blood or expressed on the cell surface. NS1 is also highly antigenic and contributes to the human antibody response repertoire against the virus [16, 17]. Recently, the NS1-based serological tests have been developed for distinguishing ZIKV from DENV infection [18–21]. But little is known about the NS1-specific Ab response during the course of the ZIKV infection with respect to its specificity, magnitude, and kinetics which are of great relevance for diagnostics [22, 23]. Here we established an IgG capture ELISA method using a recombinant full-length NS1 expressed in mammalian 293 T cells and examined the changes of NS1-specific Ab response and its cross-reactivity with four DENV serotypes in sequential plasma samples from the two Chinese travelers returning from South America where they contracted ZIKV infection. Furthermore, we isolated five NS1 monoclonal antibodies (mAbs) from these two ZIKV-infected individuals and examined the specificity of these mAbs. Finally, we investigated the binding to ZIKV NS1 in sera from a population of DENV1-infected individuals. Our results showed that a very limited cross-reactivity of human NS1 antibody response existed between ZIKV and DENV. The kinetics and specificity of NS1 Ab revealed here had important implications for NS1-based diagnostics and vaccine development.

## Methods

### Patients and blood samples

Sequential blood samples were collected from two Chinese travelers returning from South American with ZIKV infection [5, 15]. Both of them had mild clinical symptoms. Sampling time covered acute phase and early convalescent (≤100 days after symptom onset) in both individuals, with one of them being followed up to over 1 year. ZIKV infection was diagnosed as virus RNA positive by the Chinese Center for Disease Control and Prevention. The isolated plasma or PBMC was preserved at − 80 °C or liquid nitrogen tank as appropriate. Additionally, a total of 108 sera were collected on the day 6 to 260 after symptom onset between Nov 2014 and Mar 2015, from patients with RT-PCR-confirmed DENV1 infection and hospitalized during the 2014 dengue epidemic in Guangzhou The primary DENV1 infection was diagnosed as being only anti-dengue IgM positive, whereas secondary infection being positive for both anti-dengue IgM and IgG or only IgG positive using acute-phase serum samples from patients [9].

Recombinant ZIKV and DENV NS1 proteins and Western blot analysis.

The full-length NS1 coding sequences of ZIKV (KY888678), DENV1 (KY911976), DENV2 (KY911978), DENV3 (KY911981) and DENV4 (KY911982) (Additional file 1) were amplified from clinical serum samples or virus stains from infected Chinese patients by RT-PCR and cloned into the pCDNA3.1 expression vector (Invitrogen). Sequence confirmed vector was then transfected into 293 T cells and the supernatant was collected on day 4–5. The expression of ZIKV or DENV NS1 protein with the D7 tag on C-terminal in the supernatant was analyzed by Western blot using antibody D7324(Cliniqa) [24, 25]. Recombinant envelope glycoprotein of ZIKV(KU820898), DENV1(KJ438296), DENV2(JX470186), DENV3(KF824903), and DENV4(JQ822247) were produced according to the method described above with a D7-tag at the C-terminus.

### Isolation of ZIKV NS1 mAbs from memory B cells

ZIKV NS1 monoclonal antibodies were isolated from the memory B cell culture method in vitro [15]. Briefly thawed PBMCs derived from the two ZIKV-infected individuals (on day188 in patient1 and day66 in patient 2) were stained with a panel of fluorescence-labeled Abs (IgD-FITC, CD19-ECD, CD27-PC7, CD38-APCA750, IgM-PB, and CD45-KO Beckman Coulter). Memory B cells gated as IgD IgM - CD27 + CD38 low were sorted into 96-well culture plates (25 to 50 cells/well) and cultured with supplements as described previously [15]. After 10 days, the culture supernatants were screened for the presence of ZIKV NS1 binding mAbs using capture ELISA described in next paragraph. VH and VL sequences were obtained from positive B-cell cultures by RT-PCR as described [26] and analyzed using the IMGT/V-Quest program.

### Capture ELISA analysis

The capture ELISA was established as previously reported [27]. The anti-D7 antibody (D7324, Cliniqa) was pre-coated on 96-well ELISA plates. The next day, after washing the plates, culture supernatants containing the NS1 or E protein from ZIKV and DENV with the D7 tag on C-terminal were added to establish capture ELISA. The binding of serum samples or mAbs to NS1 was measured. The binding activity was detected by anti-human IgG labeled with horseradish peroxidase(HRP) and 3,3′,5,5’-Tetramethylbenzidine(TMB) substrate. Negative controls were measured by adding supernatant from the 293 T cell culture without transfection. Each sample was tested in duplicates. Absorbance value was corrected by using the mean value of negative controls.

### Statistics analysis

In ELISA analysis of NS1 binding, half-maximal effective concentrations (EC50) were calculated using the dose-response-stimulation model in GraphPad Prism (GraphPad Software Inc.). Significance analyzed by Tukey’s multiple comparisons test following two-way ANOVA.

## Results

### Expression of recombinant NS1 protein and establishment of capture ELISA

**Fig. 1:**
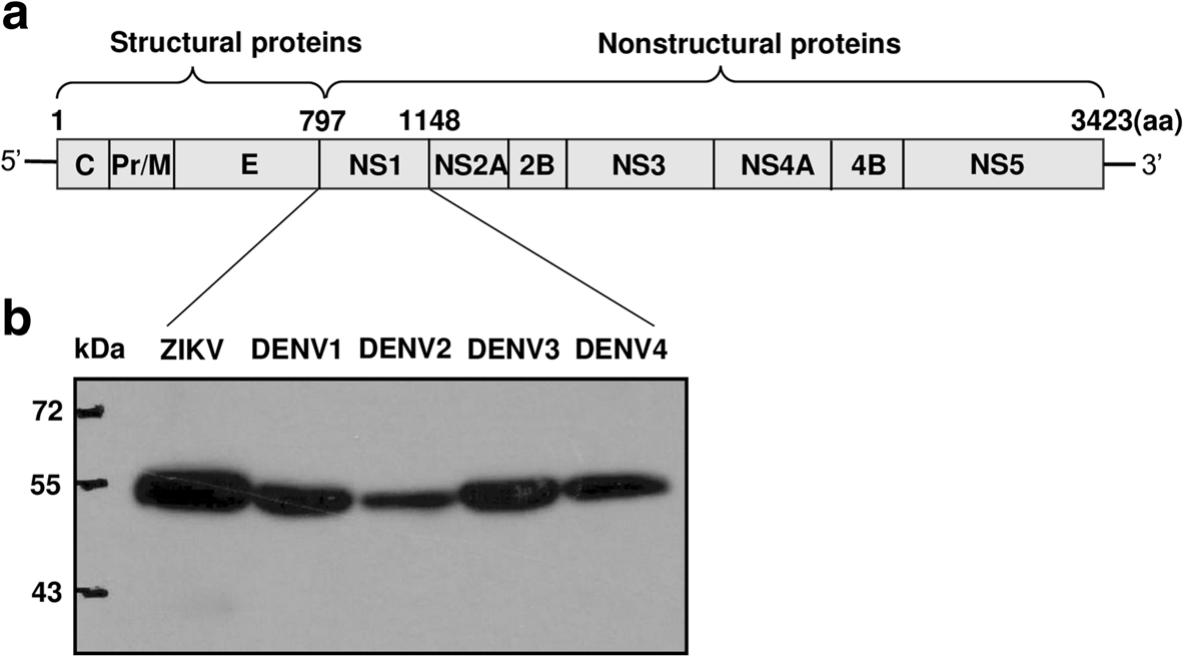
The full-length NS1 proteins of ZIKV and DENV1–4 expressed in mammalian 293 T cells. **a** The structural and non-structural proteins of flavivirus. The schematic diagram showed the length of non-structure protein NS1 was about 352aa. **b** Western blot analysis. ZIKV or DENV NS1 protein with the D7 tag on C-terminal was shown to be about 55KD as detected by the D7 antibody

To investigate the human antibody response to ZIKV NS1 protein, we first expressed the full length NS1 proteins of ZIKV and DENV1–4 in mammalian 293 T cells (Fig. 1a and Additional file 1) and collected the culture supernatants containing NS1 proteins, which were shown to be about 55KD as detected by the D7 tag antibody in western blot (Fig. 1b). These NS1 proteins were used to set up capture ELISA with D7 Ab pre-coated plates.

### Dynamics and cross-reactivity of NS1 antibody response during natural ZIKV infection

**Fig. 2:**
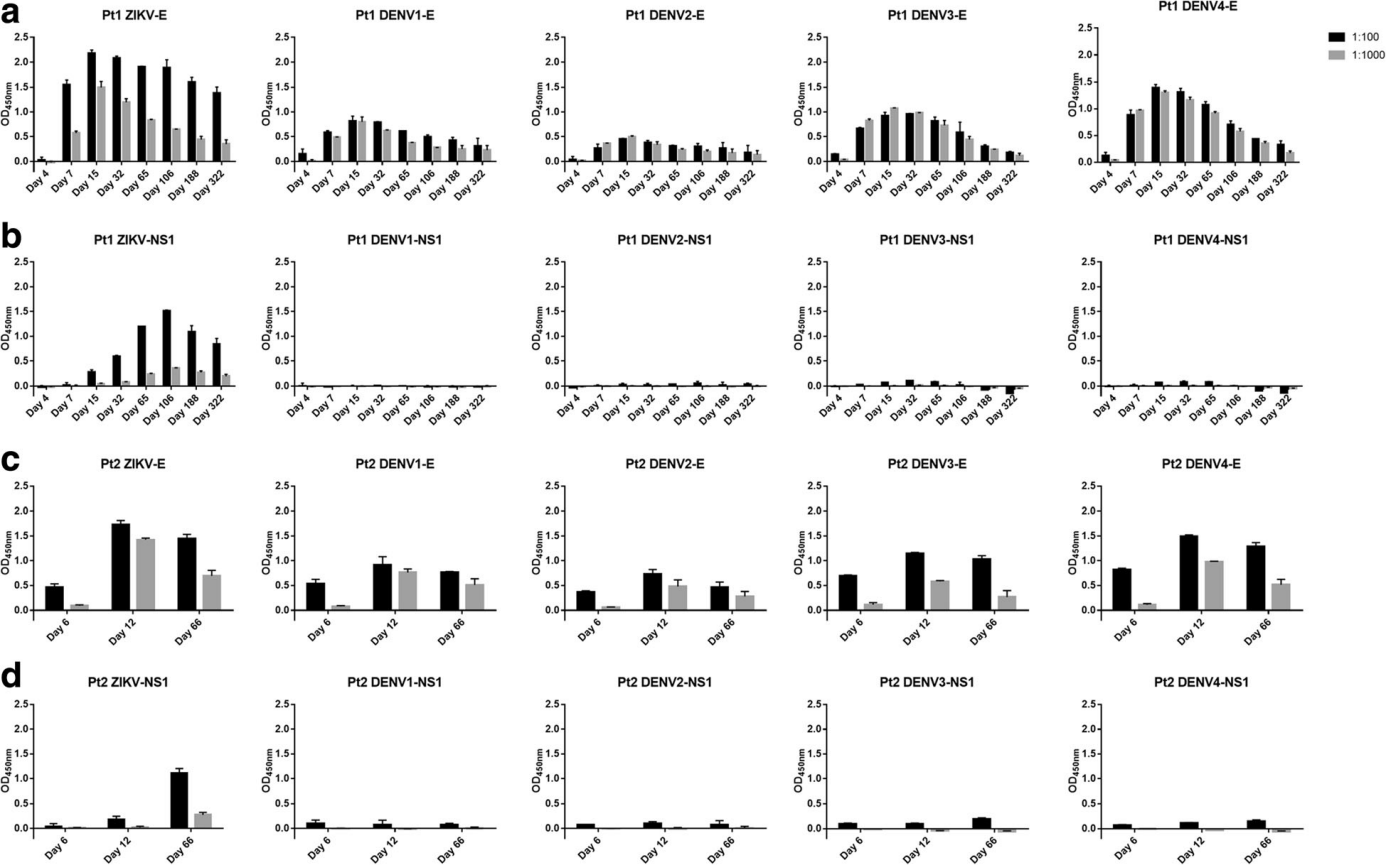
Dynamics and cross-reactivity of E and NS1 antibody responses during natural ZIKV infection. Binding of sequential plasma samples to E or NS1 of ZIKV and DENV1–4 from Pt1 (**a** and **b**) and Pt2 (**c** and **d**) were tested by ELISA at dilutions of 1:100 and 1:1000

To examine the dynamic changes of human NS1 antibody response at polyclonal level during the course of ZIKV infection, we measured the serum binding Abs in sequential samples from two ZIKV infected patients. Eight sequential plasma samples were obtained from Pt1 on day 4, 7, 15, 32, 65, 106, 188, and 322, and three were obtained from Pt2 on day 6, 12, and 66 after the onset of symptom. The binding of all these serum samples to DENV1–4 NS1 was also tested at a dilution of 1:100. Comparing to E specific Ab response reported previously in serum samples from the same patient [15] (Fig. 2a), the NS1 specific antibodies appeared later, became detectable on day 15, also peaked later on day 106 and declined markedly during disease (Fig. 2b). Similar trends of E and NS1 specific Ab responses were observed in Pt2 with peaks on day 12 and day 66, respectively (Fig. 2c and d). In contrast to high cross-reactivity of serum antibodies to DENV1–4 E proteins, there were no detectable binding of serum antibodies to DENV1–4 NS1 from both patients at all time points at a serum dilution of 1:100(Fig. 2b and d).

### Binding and specificity of NS1 mAbs derived from memory B cells of ZIKV infected patients

**Fig. 3:**
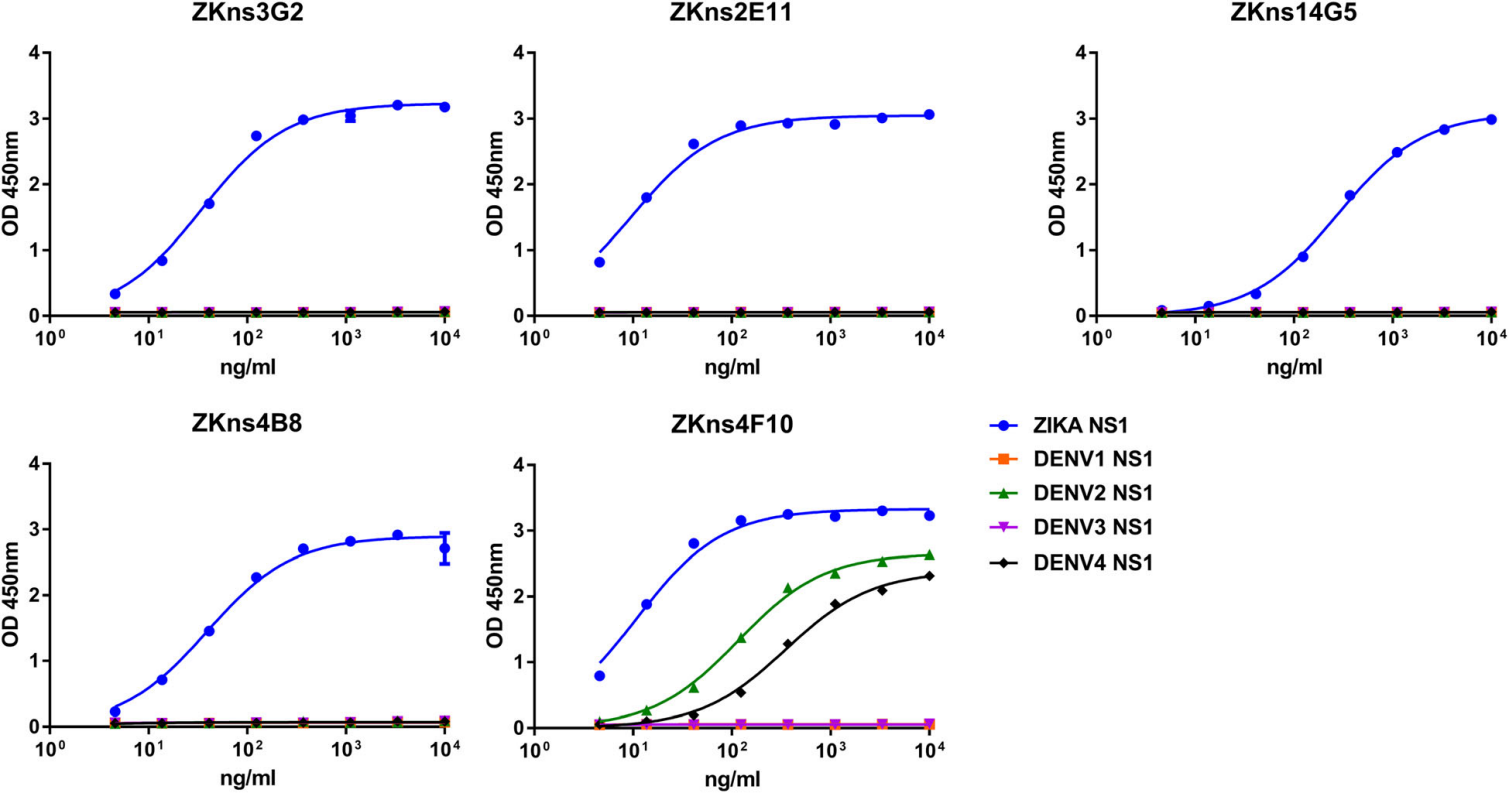
Specificity and cross-reactivity of NS1 mAbs derived from ZIKV-infected patients. Five mAbs isolated from two ZIKV donors were tested for binding to NS1 proteins of ZIKV and DENV1–4 (EC50, ng/ml). Data are representative of at least two independent experiments. The x-axis is on a logarithmic scale

To further dissect the polyclonal anti-NS1 responses, we isolated five NS1 mAbs from these two ZIKV infected patients by memory B cell culture method and characterized their CDR3 regions and pattern of reactivity (Table 1). Most of mAbs (4/5) showed strong binding to ZIKV NS1 with EC50 values ranging from 9.8 to 277.4 ng/ml, and no reactivity to DENV 1–4 NS1 proteins (Fig. 3, Table 1). Only one mAb (1/5), ZKns4F10, cross-reacted with DENV 2 and DENV 4 NS1 with EC50 of 116.1 ng/ml and 351.4 ng/ml, respectively; 10–30 fold less than that to ZIKV NS1 (10.9 ng/ml) (Fig. 3, Table 1), which can be explained by the partial sequence and structure similarity shared between ZIKV and DENV (Additional file 1).

**Table 1:** Specificity and genetic characteristics of mAbs isolated from two ZIKV infected individuals

mAb ID	Origin	Day	Binding to NS1 (EC_50_)(ng/mL)					Heavy chain			Light chain		
			ZIKV	DENV1	DENV2	DENV3	DENV4	V family	CDR3 sequence	V gene identity (%)	V family	CDR3 sequence	V gene identity (%)
ZKns3G2	Pt1	188	34.5	N/A	N/A	N/A	N/A	IGHV3–53	ARERGWLDY	93.68	IGKV1–39	QQTYTIPRT	92.83
ZKns4F10	Pt1	188	10.9	N/A	116.1	N/A	351.4	IGHV4–31	ARAIDNFYDNSI	96.56	IGKV1–39	QQSYSPPYT	94.98
ZKns2E11	Pt2	66	9.8	N/A	N/A	N/A	N/A	IGHV3–30-3	ARVFNGYEGDY	95.83	IGLV3–10	YSTDSSGNLYV	97.49
ZKns14G5	Pt2	66	277.4	N/A	N/A	N/A	N/A	IGHV5–51	ARSNVDGSTDY	98.61	IGLV3–25	QSADSSDTYVPYV	98.92
ZKns4B8	Pt2	66	39.2	N/A	N/A	N/A	N/A	IGHV3–53	ASLGSGSAFGY	96.84	IGKV1–12	QQANSFPFT	98.21

### Global analysis of NS1 cross-reactivity in DENV1 infected individuals

**Fig. 4:**
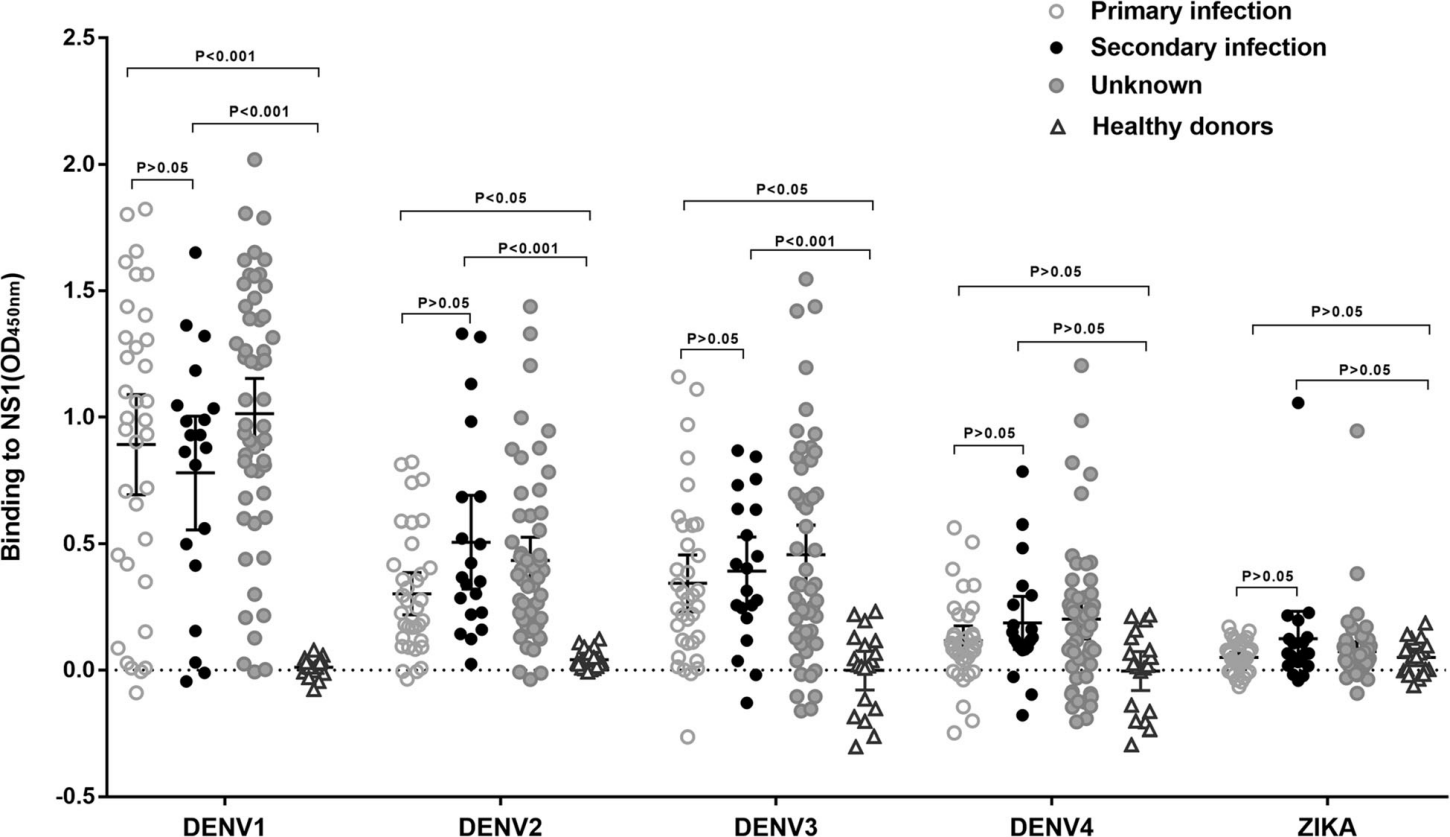
Binding of DENV1-immune plasma to recombinant ZIKV and DENV1–4 NS1 proteins. A total of 108 sera (*n* = 35 for primary infection, *n* = 20 for secondary infection and *n* = 53 for unknown) from the DENV1-infected individuals were tested by ELISA for binding to NS1 proteins of ZIKV and DENV1–4 at a 1:100 dilution. Negative controls were plasma from healthy donors (*n* = 19). Each serum sample was tested in duplicates. Significance was analyzed by two-way ANOVA followed by Tukey’s multiple comparisons test correction

To investigate whether the lack of significant cross-reactivity between anti-ZIKV NS1 antibodies and anti-DENV NS1 antibodies is unique to a few selected patients, we performed NS1 serum binding assay on a total of 108 convalescent sera from DENV1 infection including primary infection(*n* = 35), secondary infection(*n* = 20) and undetermined cases (*n* = 53) (Fig. 4). Lack of cross-reactivity to ZIKV was observed in all DENV1 infected individuals except for two cases which also exhibited significant cross-reactivity to DENV2, DENV3, and DENV4.

## Discussion

Like in DENV infection, both E and NS1 proteins are targeted by the human immune system in ZIKV infection [13, 28]. The extensive cross-reactivity between antibodies to ZIKV E protein and DENV E proteins of four different serotypes made it hard to differentiate between these two infections using the existing serology diagnostic tools. Several new NS1-based IgM/IgG ELISAs were reported for differential diagnosis of ZIKV and DENV infections [18, 21]. Therefore, the understanding of NS1-specific Ab response during natural human ZIKV infection is urgent.

Recently, the changes of ZIKV-NS1 specific IgG levels during disease was described using the commercial ELISA kits [20, 29, 30]. In contrast, we reported for the first time the detailed kinetics of both E and NS1 antibody responses during nature ZIKV infection using sequential serum samples from the early phase, convalescence phase, and up to 1 year. Compared to anti-E responses, the NS1 specific Ab responses appeared later and peaked later. Both antibody responses, however, waned over time. The trend of NS1 antibody response in our study was consistent with the latest report in which the IgG positivity peaked between day 27 and day 61 [20]. Notably, there is a marked lack of cross-reactivity in the longitude sera samples from these two ZIKV-infected individuals to DENV NS1 proteins of all serotypes, indicating they did not have a prior DENV infection history [12].

In recent publication, most of mAbs(24/30) isolated from ZIKV-infected donors without previous exposure to DENV were ZIKV-specific, whereas mAbs(5/11) isolated from DENV-immune ZIKV-infected donors were cross-reactive [12]. In our study mAbs isolated from the two ZIKV infected patients showed high specificity. Among the five mAbs, four NS1 mAbs displayed ZIKV-specific with the lowest EC50 to 9.8 ng/ml, which can be used to develop serological assays discriminating Zika virus from Dengue infection [19].

The cross-reactivity of DENV-immune sera to ZIKV-NS1 is a concern for serological test in ZIKV and DENV co-circulating areas. It was reported that 36–40% plasma samples from ZIKV-naïve DENV-immune donors including secondary DENV infections displayed cross-reactivity with ZIKV, WNV, and YFV NS1 using a direct ELISA format [19]. We observed a limited serum cross-binding to ZIKV NS1 in a population of DENV1 infected individuals, even in those with secondary DENV1 infection, only 5% (1/20) showed binding to ZIKV NS1 by the established capture ELISA method. Sera from patients infected by other DENV serotypes are under investigation for cross-reactivity to ZIKV NS1 protein. Taken together, because of the high level of cross-reactivity between ZIKV and DENV E-reactive Abs, diagnostic strategies based on NS1 Abs are a valuable tool for diagnosing ZIKV infection where DENVs co-circulate [19, 20, 31, 32].

## Conclusions

By analyzing sera obtained from patients with ZIKV or DENV1 infection, we have discovered anti-NS1 antibodies being high specific at polyclonal and monoclonal levels, this is distinct from the high cross-reactive antibody responses to ZIKV and DENV E proteins. This observation supports the development of NS1 based diagnostic tools for areas where DENV and ZIKV co-circulate.

## Additional file

Additional file 1: Sequence alignment of NS1 proteins of ZIKV and DENV1–4. Overall amino acid identity of each of the four DENV serotypes to ZIKV was shown on the top. Conservative regions were highlighted in color. The amino acid sequences encoding β-roll domain (1-30aa), wing domain (31-180aa), and β-ladder domain (181-352aa) were shown. (TIFF 872 kb)

## Abbreviations

Abs: Antibodies
CDR3: Complementarity-determining region 3
DENV: Dengue virus
DENV1: Dengue virus serotype 1
DENV2: Dengue virus serotype 2
DENV3: Dengue virus serotype 3
DENV4: Dengue virus serotype 4
E: Envelope protein
EC50: Concentration for 50% of maximal effect
ELISA: Enzyme-linked immunosorbent assay
FLE: Fusion loop epitope
IgG: Immunoglobulin G
IgM: Immunoglobulin M
mAbs: Monoclonal antibodies
NS1: Non-structural protein 1
WHO: World Health Organization
ZIKV: Zika virus
