# Supplementary figures and images for: Delayed and highly specific antibody response to nonstructural protein 1 (NS1) revealed during natural human ZIKV infection by NS1-based capture ELISA
Source: BMC Infect Dis. 2018 Jun 14;18:275. doi: 10.1186/s12879-018-3173-y (PMC6000977; doi:10.1186/s12879-018-3173-y)

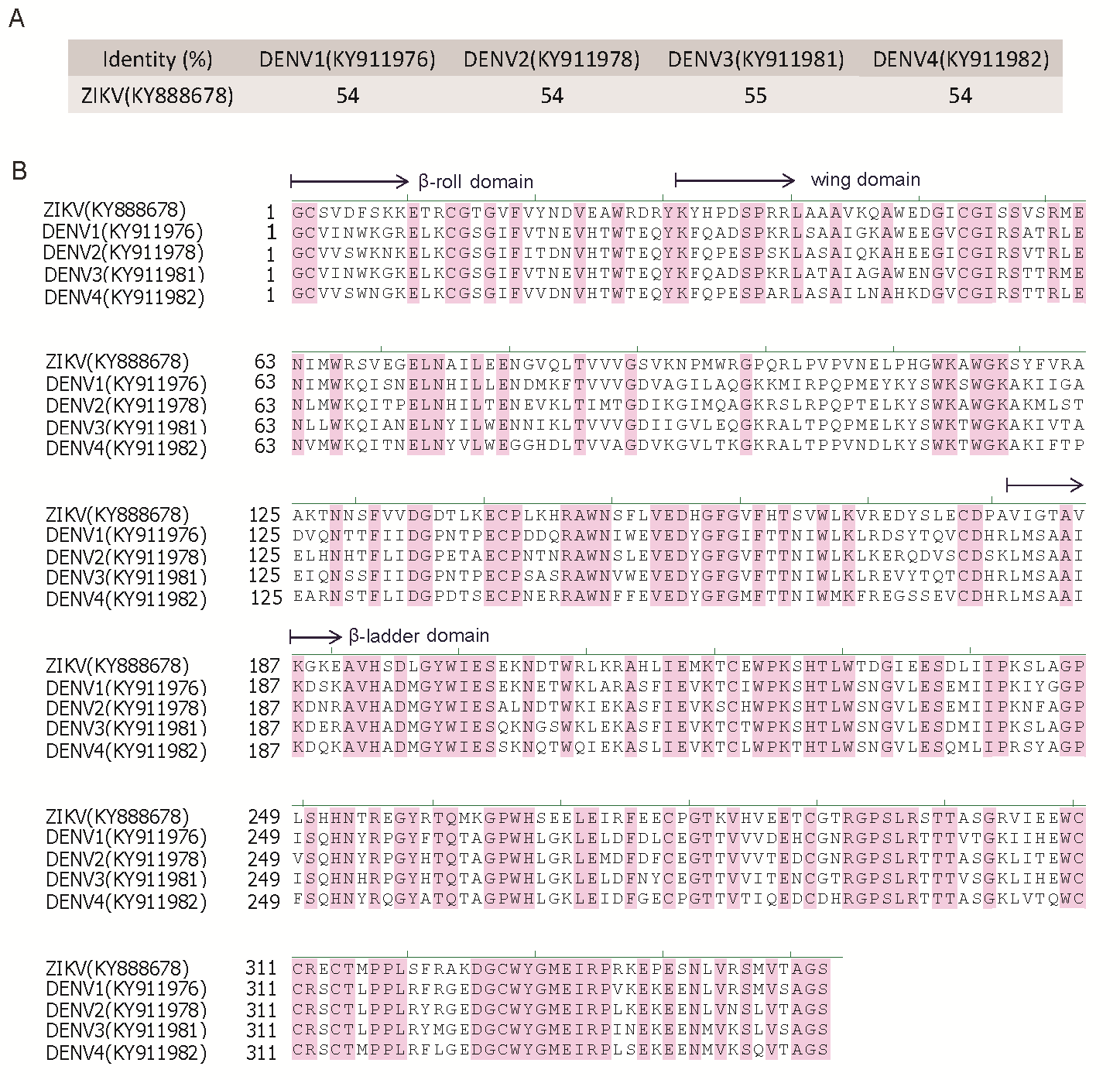

Supplement: Supplementary file 1 — Sequence alignment of NS1 proteins of ZIKV and DENV1–4. Overall amino acid identity of each of the four DENV serotypes to ZIKV was shown on the top. Conservative regions were highlighted in color. The amino acid sequences encoding β-roll domain (1-30aa), wing domain (31-180aa), and β-ladder domain (181-352aa) were shown. (TIFF 872 kb) [file 12879_2018_3173_MOESM1_ESM.tiff]
